# A new method of endoscopic ultrasound and cholangioscopy-guided gastroenterostomy

**DOI:** 10.1055/a-2610-3021

**Published:** 2025-06-26

**Authors:** Cancan Zhou, Zheng Wang, Zheng Wu, Hao Sun, Jie Hao

**Affiliations:** 1 162798 Department of Hepatobiliary Surgery, The First Affiliated Hospital of Xiʼan Jiaotong University, Xiʼan, China

Endoscopic ultrasound (EUS)-guided gastroenterostomy (EUS-GE) using lumen-apposing metal stents (LAMS) has emerged as a promising minimally invasive approach for managing malignant gastric outlet obstruction 1 .

A 67-year-old man with pancreatic carcinoma presented with symptoms of gastric outlet obstruction. He was scheduled for EUS-GE under direct cholangioscopic guidance without X-ray. A 9-Fr cholangioscope (Micro-Tech, Nanjing, China) was advanced transnasally into the stomach and traversed the pylorus ( Fig. 1 ). Under the guidance of a 0.035-inch guidewire, the cholangioscope navigated through the stenotic duodenal bulb and distally to the jejunum ( Fig. 2 ). Simultaneous EUS imaging confirmed the location of the cholangioscope and dilated jejunum by water injection ( Fig. 3 ). A 20-mm LAMS (Hot AXIOS stent; Boston Scientific, Marlborough, Massachusetts, USA) was deployed through the gastric wall into the jejunum. Expansion of the distal flange was visualized in the cholangioscopic and EUS views. The electrocautery tip and the correct positioning and expansion of the distal flange in the jejunum were confirmed ( Fig. 4 ). Following deployment of the proximal flange, cholangioscopic methylene blue injection confirmed unimpeded stent patency with dye passage into the gastric lumen. After dilation of the LAMS, EUS-GE was completed without X-ray ( Video 1 ).

**Fig. 1:**
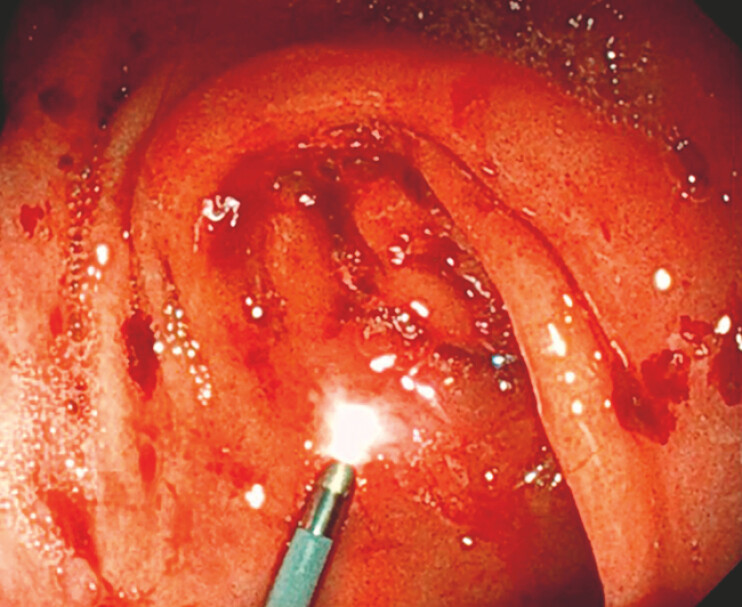
The cholangioscope was passed transnasally into the stomach and traversed the pylorus.

**Fig. 2:**
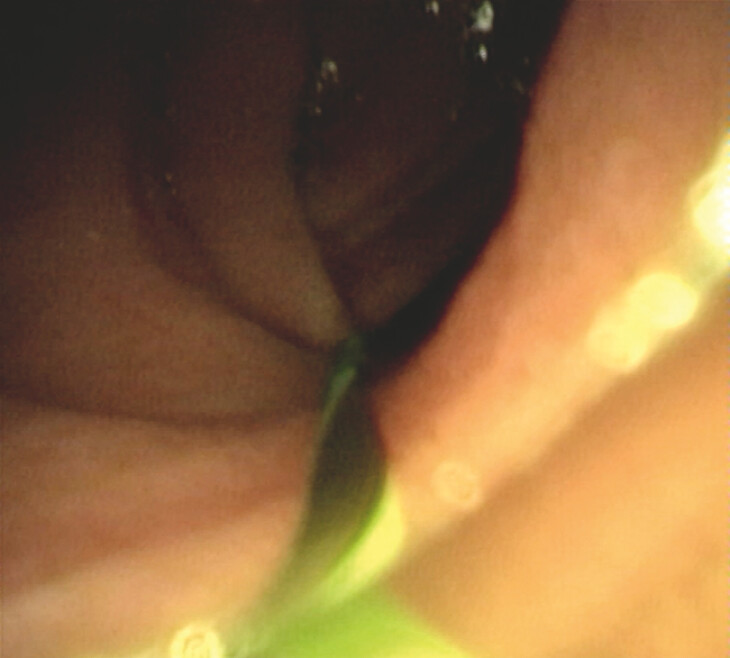
The cholangioscope passed through the obstruction into the jejunum under wire guidance.

**Fig. 3:**
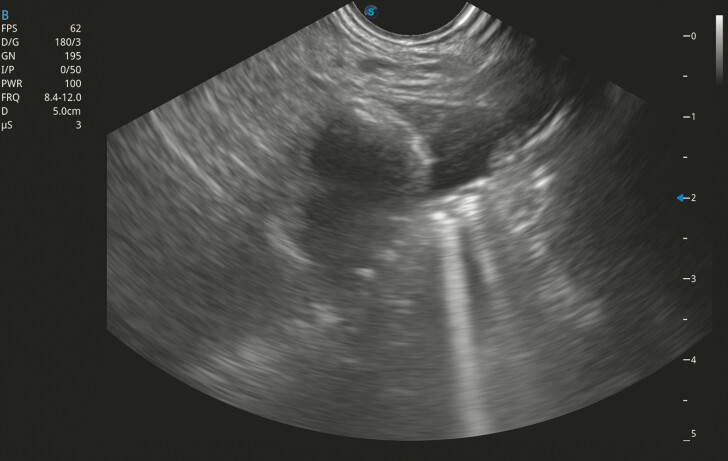
Endoscopic ultrasound visualization showed the dilated jejunum and the scope sign.

**Fig. 4:**
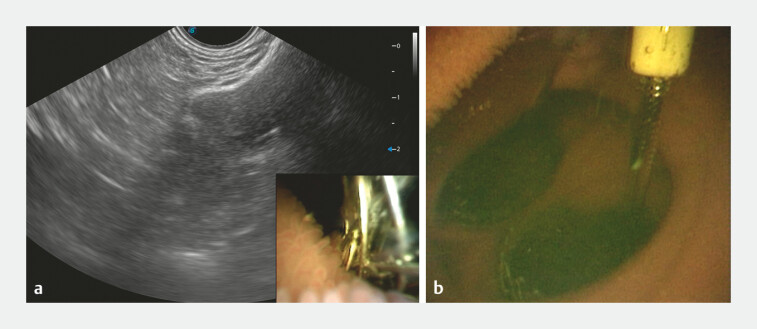
Confirmation of the position of the distal flange and electrocautery tip. **a** Expansion of the distal flange was visualized both in cholangioscopic and endoscopic ultrasound views. **b** The electrocautery tip was seen in the jejunum.

The cholangioscope was advanced to the distal jejunum and expansion of the distal flange was confirmed by both cholangioscope and endoscopic ultrasound. Then, the gastroenterostomy was performed using a lumen-apposing metal stent without X-ray.Video 1

An upper gastrointestinal contrast study confirmed the patency of the stent ( Fig. 5 ). The patient tolerated a liquid diet and was discharged on postoperative day 4, with sustained clinical improvement.

**Fig. 5:**
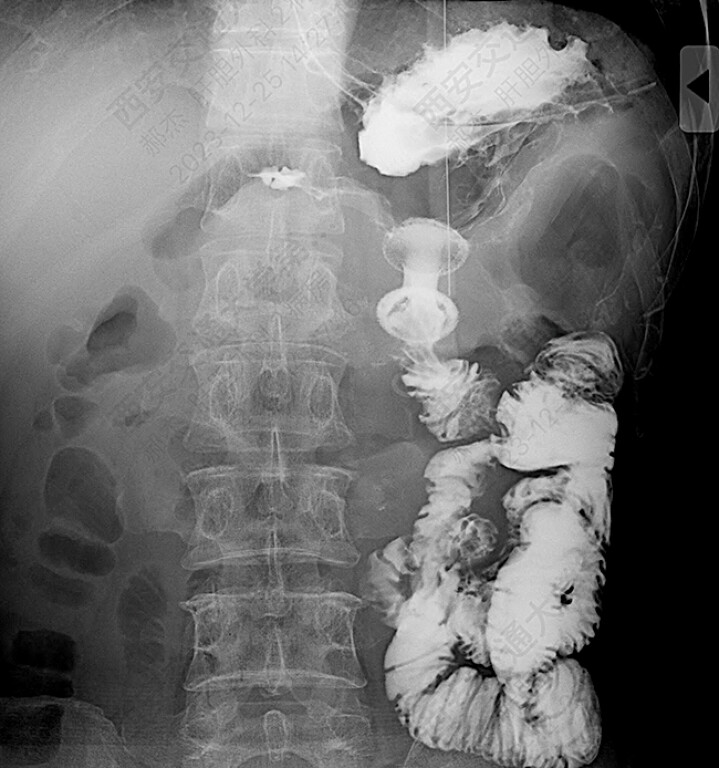
Postoperative upper gastrointestinal radiography confirmed the patency of the stent.

Currently, EUS-GE techniques such as the balloon-occluded method and wire-guided electrocautery-enhanced stent placement (WEST) method aim to simplify the procedure, but the risks of misdeployment or inadvertent colonic anastomosis remain 2 3 . The WEST technique, despite its procedural streamlining, still reports adverse events including bleeding and perforation 4 . In contrast, our cholangioscopy-guided approach offers two key innovations. First, enhanced precision: cholangioscopic guidance allows immediate verification of LAMS deployment, reducing the risk of misplacement or collateral injury to adjacent organs. Second, real-time anatomic confirmation: direct cholangioscopic visualization eliminates reliance on fluoroscopy, mitigating radiation exposure for both patients and operators. This is particularly critical in minimizing cumulative radiation risks in recurrent procedural settings.

Endoscopy_UCTN_Code_TTT_1AS_2AG

## Notes

Endoscopy E-Videos https://eref.thieme.de/e-videos
*E-Videos* is an open access online section of the journal *Endoscopy* , reporting on interesting cases and new techniques in gastroenterological endoscopy. All papers include a high-quality video and are published with a Creative Commons CC-BY license. Endoscopy E-Videos qualify for HINARI discounts and waivers and eligibility is automatically checked during the submission process. We grant 100% waivers to articles whose corresponding authors are based in Group A countries and 50% waivers to those who are based in Group B countries as classified by Research4Life (see: https://www.research4life.org/access/eligibility/ ). This section has its own submission website at https://mc.manuscriptcentral.com/e-videos .
